# Objective vs. Subjective Health in Very Advanced Ages: Looking for Discordance in Centenarians

**DOI:** 10.3389/fmed.2018.00189

**Published:** 2018-06-26

**Authors:** Lia Araújo, Laetitia Teixeira, Oscar Ribeiro, Constança Paúl

**Affiliations:** ^1^Center for Health Technology and Services Research, Institute of Biomedical Sciences Abel Salazar (CINTESIS-ICBAS), University of Porto, Porto, Portugal; ^2^School of Education, Polytechnic Institute of Viseu, Viseu, Portugal; ^3^Department of Education and Psychology, University of Aveiro, Aveiro, Portugal

**Keywords:** centenarians, longevity, physical health, self-rated health, well-being paradox

## Abstract

**Background:** Living beyond 100 years of age is associated with several functional and health constraints but their impact depends on one's perception of the situation. Associations between self-rated health (SRH) with sociodemographic and psychosocial variables have been explored in several studies, revealing that one's health appraisal depends of factors beyond the objective health condition. There is a large body of literature concerning SRH in later life but lack of evidence about centenarians' perception of health and its associated factors, which could increase the available knowledge on the strengths and resources individuals in very advanced ages have for facing daily life limitations.

**Objective and Methods:** This study aims to analyse the relationship between subjective and objective health status in a sample of centenarians (*n* = 127). Subjective health was assessed by a single-item health measure, and objective health by considering the number of reported diseases and a functional capacity scale (BADL and IADL). Main health characteristics are described as well as examined the association between objective and subjective health.

**Results:** 46.5% of the sample has a good, very good, or excellent appraisal of their own health. SRH was associated (*p* < 0.05) with BADL and IADL scores and with the total number of diagnosis; when analyzing SRH according to the level of functional capacity, results revealed that most individuals with severe and moderate dependence have a reasonable to excellent SRH (*p* > 0.05).

**Conclusion:** Having diseases and functional dependence at 100 years old may not mean to have a bad SRH. The high variability in SRH and the discordance between objective and subjective measures are a proof of centenarian's capacity of adaptation and the existence of individual resources, which may be decisive for one' perception and handling of health situation at such an advanced age.

## Introduction

One of the great achievements of the Twenty-first century will undoubtedly be the increase in exceptional longevity. As projected by the Population Division of the United Nations (1), at the beginning of 2100 there will be more than 25 million of individuals reaching 100 years old, which is more than 50 times the size of the population of centenarians in 2015. Japan is the leading country in terms of number of centenarians, but also in Europe, due to low fertility rates and decreasing old age mortality, there is an emergence of oldest old groups (2). In 2011, the total number of centenarians in Europe was 89,156, with France, Italy, and Greece presenting ratios higher than 20 centenarians per 100,000 inhabitants (3).

Gerontology and geriatric research has been affirming the importance of distinguishing various age subgroups within old age, in the sense that there are striking differences among young-old and the old-old or very-old, i.e., between the third and the fourth ages (4). Centenarians' studies have been revealing that reaching 100 years old brings several challenges at an individual level, but also to formal and informal carers since age-related adversities and age-related needs can be particularly demanding. Functional decline (e.g., physical health/activities of daily living restrictions, mobility, sensory impairment), and psychological and social losses (e.g., loss of loved ones and appreciated activities, dependency have been reported as being often present in such an advanced ages (5, 6).

As long life and longevity increases, an interest toward positive aspects of aging is gaining strength. Due to the difficulty in preventing and avoiding major age-related constrains, there is an increased investment in knowing how individuals manage adversity and in discovering the resources and protective factors of positive functioning (7). The ability to maintain positive self-perceptions and well-being despite hardships is considered an important paradox in advanced life, closely related with one's psychological resilience (8).

Self-rated health (SRH) is one of the most recognized subjective measures in aging research, clinical settings, and population's surveys. With a single and simple question (“In general, would you say your health is…”) it is possible to know if the person considers his/her health as “excellent,” “very good,” “good,” “fair,” or “poor” (9, 10). The importance of this question also relates to its predictive value and independent effect on mortality, which has been demonstrated in numerous studies and diverse populations (11, 12).

Despite being considered a measure to examine subjective health status, several studies have shown that SRH reveals much more information. SRH is related to disease and functional status (13), but also with mental health [e.g., depression; (14)], suggesting that SRH can reflect the states of the human body and mind (12). Also psychosocial variables (e.g., life satisfaction and social support) and socioeconomic status have shown correlation with SRH (11, 15, 16), as well as personality traits (17) and other internal (e.g., as optimism and perceived control) and external (e.g., education, financial status) resources (18).

Due to the observed relation between SRH and objective health aspects, a decrease of SRH with advancing age is to be expected. However, evidence does not irrefutably confirm this hypothesis. Two reasons have to be considered. First, the relationship between subjective and objective health is complex and not direct or independent. Second, in old and very-old age there is a paradoxical pattern of discrepancy between subjective and objective indicators of health. Although there are some contradictions across studies, it seems that SRH does not decline only due to age-related decrease in health status (19). In Pinquart's Pinquart's (10) meta-analysis, for instance, there was a larger association with physical illness and functional limitations in those aged 60–75 years when compared to those aged above 75. Also in the oldest group, an increased association between mental health and SRH was verified (10).

Henchoz et al. (19), French et al. (14), and Zikic et al. (20), have studied objective and subjective measures of health in individuals with 80, 85, and 90 plus years old, respectively. Their findings point to a weaker relationship between objective health measures (e.g., medical diagnoses) and SRH, and to a less rapidly decline of the perception of health with advancing years than the one occurring in physical and functional health status. This weakening relationship between subjective and objective measures of health can be accounted for by several factors, including the capacity to adapt and be resilient, and can be an important indicator of the presence or absence of external and internal resources that might influence life's appraisal.

Despite some differences across studies, the available evidence on the oldest-old show that is possible to reach 100 years old in a relatively good health condition (21, 22). Centenarians have been presented as robust and resistant individuals, since they tend to survive, delay, or escape to the major age-related diseases, such as cancer, and cardiovascular diseases among others (23, 24). But there are many centenarians living in a frail and morbidity situation as well (25, 26). Also great difficulties in sensory domains and basic and instrumental daily living activities (BADL and IADL) have been widely reported in this population [cf. (27–29)]. Nevertheless, several investigations are looking to this age group as a prototype of successful aging (30, 31) due to their ability to maintain a positive outlook about life.

Centenarians can be a very interesting group to examine objective vs. subjective health appraisals because they have to face several health and functional capacity problems. The study of this relation may reveal if SRH can serve as an indicator of centenarians' objective health status and if the discrepancy between the two dimensions of health still exists in such an advanced age. In this study, we sought to examine the association between centenarians' own subjective evaluations of health and their objective health status.

## Methods

### Data collection

Data came from two centenarians studies, the Oporto Centenarian Study (PT100) and the Beira Interior Centenarian Study (PT100 Beira Interior) which were conducted in two distinct geographical regions of Portugal, each one with an area of approximately 60 km. Individuals aged 100 years and older between December 2013 and December 2014 were identified through voter registration files, churches, nursing homes, local media newspapers, and through snowball sampling. This first step of recruitment resulted in 291 potential participants; all of these were contacted, and a final sample of 241 participants was face-to-face interviewed. Fifty centenarians were excluded because they died in the interim or their relatives refused participation because of advanced dementia and other major health problems or due to lack of interest in the study. Since this study requires centenarians' own perceptions, information was only assessed if the individual was not affected by severe cognitive impairment and was willing to present information on these aspects (*n* = 127).

Data was collected during one or two sequential interview sessions directly with the centenarian and/or with a proxy respondent. Age was verified by following a protocol entailing personal identity document verification (e.g., birth certificate) and milestones assessments (e.g., wedding date, date of firstborn, subsequent birthdates of children) following best research practices in this field (32). An informed consent previously approved by the National Commission on Data Protection was used. More information about the methodological procedures of both centenarian studies can be found elsewhere (33).

#### Measures

Three variables were considered as objective health: the number of diseases, the functional capacity in basic activities of daily living (BADL) and in instrumental activities of daily living (IADL). Diseases were assessed with a list of common health problems in older ages: high blood pressure, heart condition, diabetes, chronic lung disease, ulcers or other serious stomach issues, cirrhosis or other liver problems, kidney condition, frequent urinary infections, incontinence, prostate problems, problems with vision or hearing, arthritis, osteoporosis, stroke, cancer, pneumonia, falls, and other. Conditions mentioned as “other” were later coded. Functional disability was assessed through the Older Americans Resources and Services (OARS) Multidimensional Functional Assessment Questionnaire (34, 35). The scale includes SEVEN items to assess basic daily living activities (BADL, e.g., the ability to talk on the phone, to travel, go shopping, prepare meals) and other seven items to evaluate IADL (e.g., the capacity for walking, bathing, eating, toileting). Respondents were asked how much difficulty they had performing each of these activities by rating them on a three-point scale (2 = no difficulty; 1 = do with some help; 0 = cannot do without help). Cronbach's alpha for this study was 0.909 for the BADL scale and 0.879 for the IADL scale. Information regarding these three variables was collected with the centenarian's proxies, in most cases a family member or in the case of institutionalized centenarians it was a professional (e.g., nurse) of the nursing home.

Self-rated health (SRH) was assessed directly with the centenarian through a single item: “In general, would you say your health is…?,” with five response options labeled as excellent, very good, good, reasonable, and bad. Responses were scored in 1 indicating a bad SRH, 2 for a reasonable SRH, and 3 for a positive SRH (excellent, very good, and good).

According previous work (36, 37), BADL, IADL, and number of diagnoses were categorized in three categories each. For ADL, the categories considered were: 1—Mild (IADL dependence only); 2—Moderate (dependent in 1–2 BADL); 3—Severe (dependence in 3 or more BADL). For the number of diagnoses, the three categories considered were: 0–1; 2–3; ≥4. Additionally, SRH was also considered as a three-point scale: 1—bad; 2—reasonable; 3—good, very good, or excellent.

Sociodemographic data was obtained from structured questions about age, gender, current marital status, living arrangements, having children, income per month, and income management.

### Statistical analysis

Description of the sample was performed using frequencies (absolute and relative), mean and standard deviation. Mean differences of objective health measures according to self-perception of health were performed considering a one-way ANOVA. To evaluate the association between categorical variables (objective health and SRH), Chi-square test was used. In all analysis, a significance level of 0.05 was considered.

## Results

### Sample characteristics

The sample comprises 127 centenarians with a mean age of 101.1 years (sd = 1.5 years, range = 100–108). One hundred and twelve centenarians are female (88.2%) and only 15 are male (11.8%). The majority are widowed (*n* = 108, 85.0%), 13 (10.2%) never married, 5 (3.9%) are married, and only 1 (0.8%) is divorced. Forty-six (36.2%) lived in an institution, and 12 (9.4%) lived alone. One hundred and seven (84.3%) have children. Almost 50% of the sample never attended school (*n* = 59, 46.5%). Concerning income, 20 (16.9%) receive <250 €/month, 78 (66.1%) receive between 250 € and 500 €, 16 (13.6%) between 500 € and 750 €, and 4 (3.3%) more than 750 €. Forty-nine (40.5%) reveal that cannot make ends meet, 52 (43.0%) just manage to get by, 14 (11.6%) have enough money with a little extra, and only 6 (5.0%) refer that money is not problem (Table 1).

**Table 1:** Sample characteristics.

	***N***	***n***	**%**
**Gender**	127		
Male		15	11.8
Female		112	88.2
**Age**			
Mean (sd)	127	101.1 (1.5)	
**Marital status**	127		
Never married		13	10.2
Married		5	3.9
Divorced		1	0.8
Widowed		108	85.0
**Living arrangements**			
Live in a institution	127	46	36.2
Live in community	127	81	63.8
**Have children**	127	107	84.3
**Attended school**	127	68	53.5
**Income per month**	118		
<250 €		20	16.9
250–500 €		78	66.1
500–750 €		16	13.6
750–1,000 €		1	0.8
>1,000 €		3	2.5
**Income management**	121		
Can't make ends meet		49	40.5
Just manage to get by		52	43.0
Enough money with a little extra		14	11.6
Money is not a problem		6	5.0

### Subjective and objective health

Fifty-nine centenarians (46.5%) report their health as good, very good or excellent, 48 (37.8%) as reasonable, and 20 (15.7%) as bad (Table 2). Concerning objective health, the mean score of BADL and IADL is 8.6 (sd = 4.2) and 3.8 (sd = 3.3), respectively. The average number of diagnoses reported by the centenarians is 3.6 (sd = 2.0), ranging from 0 (minimum) and 9 (maximum).

**Table 2:** Subjective and objective health of the sample.

	***N***	***n***	**%**
**Self-Rated Health** (SRH)	127		
Bad		20	15.7
Reasonable		48	37.8
Good, very good, or excellent		59	46.5
**BADL**, mean (sd)	127	8.6 (4.2)	
**IADL**, mean (sd)	127	3.8 (3.3)	
**Number of diagnosis**, mean (sd)	127	3.6 (2.0)	

### Association between self-perception of health and physical and functional health

**Figure 1:**
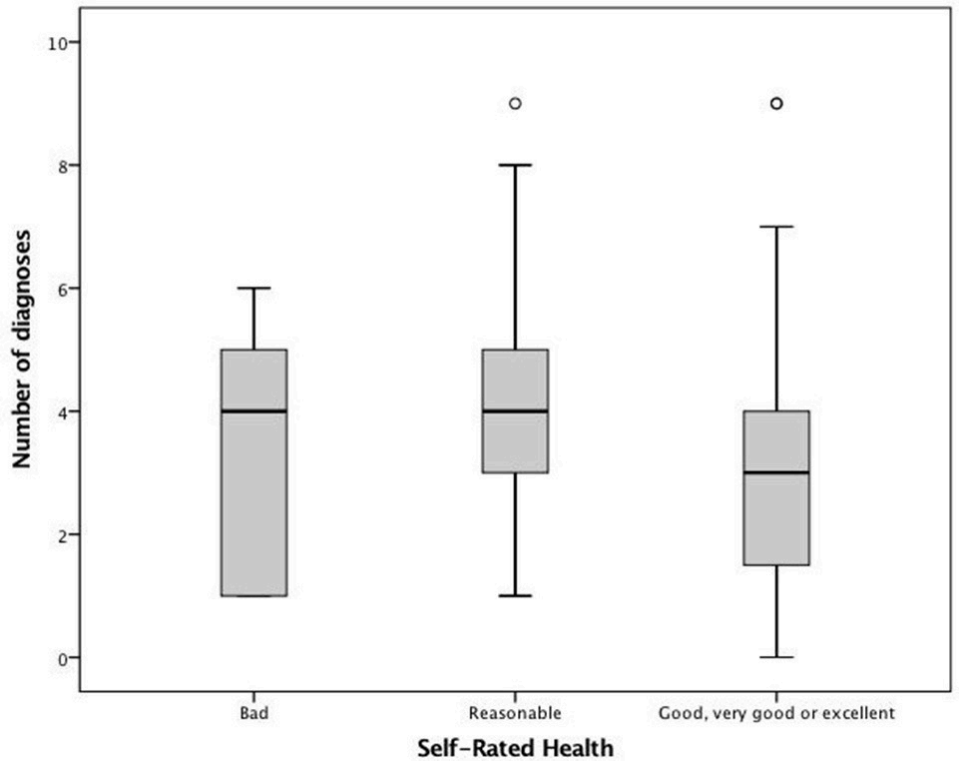
Association between SRH and the number of diseases.

Considering the three objective measures of health (continuous variables), and comparing the mean values according to the three groups of SRH, we can verify in Table 3 that differences between groups were found (*p* < 0.05 for the three variables). Both BADL and IADL scores increase with the improvement of self-perception of health (i.e., better functional capacity was related with better SRH). Considering the number of diagnoses, results revealed that the group with a reasonable self-perception of health presented a higher number of diseases than the group with a bad self-perception (Figure 1).

**Table 3:** BADL, IADL, and number of diagnoses (mean scores) according to SRH.

	**Bad SRH**	**Reasonable SRH**	**Good, very good or excellent SRH**	***p***
	**Mean (sd)**	**Mean (sd)**	**Mean (sd)**	
BADL	6.46 (3.73)	8.40 (4.23)	9.57 (4.03)	0.013
IADL	2.20 (2.34)	3.50 (2.73)	4.65 (3.76)	0.010
Number of diagnosis	3.40 (1.90)	4.17 (1.85)	3.22 (2.02)	0.041

The Table 4 presents the results obtained (the percentage presented are % of total). No significant association was found between ADL and SRH. The percentage of centenarians with some extent of agreement between the two measures was 42%. Additionally, 29% referred a worse SRH comparing with the ADL capacities, and 29% referred a better SRH comparing with the ADL capacities. A significant association was found between SRH and number of diagnoses. The percentage of some agreement of the two measures was 32.3%. Only 8.7% referred a worse SRH comparing with the number of diagnoses and 59% referred a better SRH comparing with the number of diagnoses.

**Table 4:** Association between SRH and objective health measures (categorical variables).

	**Bad SRH**		**Reasonable SRH**		**Good, very good or excellent SRH**		***p***
	***n***	**%**	***n***	**%**	***n***	**%**	
ADL							0.264
Severe (dependence in 3 or more BADL)	8	6.5	14	11.3	12	9.7	
Moderate (dependent in 1–2 BADL)	4	3.2	8	6.5	10	8.1	
Mild (IADL dependence only)	6	4.8	26	21.0	36	29.0	
Number of diagnosis							0.006
≥4	12	9.4	31	24.4	15	17.3	
2–3	2	1.6	14	11.0	22	17.3	
0–1	6	4.7	3	2.4	22	11.8	

## Discussion

The present study analyzed different aspects of health in a sample of Portuguese centenarians, comparing their perceived health with measures of objective health status. Centenarians presented a mean number of diseases of 3.6, as well as several functional limitations. These results are in line with other international studies conducted with centenarians, such as the ones from Georgia and Fordham (USA), Denmark and Heidelberg (Europe) (6, 25, 28, 29). Nevertheless, almost half (46.5%) of our sample perceived their health positively (good/very good/excellent). This percentage is, however, lower than the results presented by Cho et al. (30), Jopp et al. (29), Tigani et al. (38), and Liu and Zhang (15) who revealed that 73, 67, 66.8, and 54.3% of the centenarians in their studies rated their health in similar positive ways. These results can be understood under the influence of age and culture in self-ratings of health (12).

When examining the association between centenarians' own perception of health and their functional capacity, results of our study revealed that in overall BADL and IADL scores are associated with SRH, but that when further analyzing the different levels of dependence according to SRH, the association is no longer statistically significant. The majority of centenarians with mild dependence (IADL dependence only) have a positive (good, very good, excellent) SRH, but also the centenarians with moderate and severe dependence have higher rates of reasonable and good-excellent SRH (rather than a poor SRH). The association between the number of diagnosis and the SRH is statistically significant and the pattern is very similar to the previous one. The majority of centenarians with zero or one disease have a positive SRH, but the same happens for the ones with two or three diseases. Even the majority of centenarians with four or more diseases have a reasonable or good-excellent SRH. Together, these results allow us to state that having more diseases and dependence is not necessarily a sign of having a bad or negative SRH.

As verified with very old individuals [e.g., (10, 14, 17, 39)], it seems that also centenarians hold a weaker emphasis on their physical and functional status in the appraisal of their health condition. Previous studies that have shown discordance between objective and subjective health measures have associated it with the contribution of external and internal resources as optimism and perceived control (18, 40). A positive SRH may reflect the greater importance of psychological adaptation in very advanced age. Also the influence of downward social comparison has been reported. It seems that comparing oneself with others from the same age group who are in poorer health enables oldest old individuals (aged 80 plus) to maintain a positive SRH (19, 25). This is an important and common mechanism for the oldest old, since it is more frequent to find congeners in poor health at the age of 85 than at the age of 20, 40, or even 60 (19); in the cases of centenarians, however, since most individuals of the same generation are already dead, the comparison may elicit a more positive appreciation of one's health as it focuses on the exceptionality of still being alive. To date, there is limited information on the social comparison processes underneath SRH at such an advanced age, particularly the age group target for comparisons and its consequences for well-being. Such psychosocial process deserves, therefore, further attention.

Due to the great individual differences and disparities among oldest old individuals, especially in self-appraisals, several researches have been considering the role of risk and protective factors in explaining such variability. These studies have been presenting as a common trait of this age group the high weight of mental health aspects and psychosocial well-being factors when considering the correlates of SRH (10, 14, 17, 38). Puvill et al. (40) when analyzing the correlates of SRH in a representative population of 85-year olds found a weaker association with mortality and a stronger with mental health and life satisfaction. Therefore, an underrated subjective health condition may be indicative of psychosocial distress or burden of physical disability (14) and these health pessimists may be prone to depressive feelings (39). SRH has also been linked to frailty and anxiety in centenarians. Ribeiro et al. (26), for instance, found that SRF was the only predictor of depression in frail and pre frail centenarians and that a worse SRH increased the odds of experiencing clinical anxiety (41). When comparing centenarians with sexagenarians and octogenarians, Quinn et al. (42) found personality and levels of control as unique set of SRH's correlates in the oldest-old group. An apprehensive personality and low levels of control over health were more important than physical health variables in predicting a poor subjective health. Also Ruthig and Chipperfield (43), in a study on health incongruence in later life (ages 79–98 years) found that perceived control was weaker among pessimists; Tigani et al. (38) in a sample of Greek centenarians found that high optimism, adaptability and internal health locus of control were independently associated with good SRH.

## Conclusion

The analysis of associations between objective and subjective health allowed to conclude that not all centenarians with moderate/severe constrains of ADL and diseases have a bad SRH, which may be related with the existence of other factors that are weighted in one's self-perception of health at such and advanced age. Being in the limit of longevity brings several challenges at the health level, and these may demand specific developmental regulation processes for fostering well-being. Research on these long-lived individuals regarding their difficulties but also the variables promoting resilience (and inherent positive SRH) is a necessary investment in order to reach better years of life. Future studies should further examine the association between objective and subjective measures of health by considering the influence of adaptive resources, such as characteristics of personal disposition, and protective social comparison mechanisms which may be decisive for centenarians' perception and handling of health limitations.

## Ethics statement

This study was carried out in accordance with the recommendations of the Instituto de Ciências Biomédicas Abel Salazar (Universidade do Porto, Portugal) with written informed consent from all subjects. All subjects gave written informed consent in accordance with the Declaration of Helsinki. The protocol was approved by the Portuguese national data protection commission.

## Author contributions

OR, LA, and LT were responsible for the study conception and design. OR supervised data collection and helped writing the manuscript. LA wrote the manuscript. LT performed the data analysis and CP critically revised the paper for important intellectual content.

### Conflict of interest statement

The authors declare that the research was conducted in the absence of any commercial or financial relationships that could be construed as a potential conflict of interest.
